# Reply to Vera Osipova et al.

**DOI:** 10.1007/s10194-012-0451-8

**Published:** 2012-04-25

**Authors:** Gian Camillo Manzoni, Vincenzo Bonavita, Gennaro Bussone, Pietro Cortelli, Maria Carola Narbone, Sabina Cevoli, Domenico D’Amico, Roberto De Simone, Paola Torelli

**Affiliations:** 1 Department of Neurosciences, Headache Centre, Istituto di Neurologia, University of Parma, c/o Azienda Ospedaliero-Universitaria (Padiglione Barbieri, 3° piano), Via Gramsci 14, 43100 Parma, Italy; 2 University “Federico II” of Naples, Naples, Italy; 3 Clinical Neurosciences Department, C. Besta National Neurological Institute, Milan, Italy; 4 Department of Neurological Sciences, University of Bologna, Bologna, Italy; 5 Department of Neurosciences, Psychiatric and Anesthesiological Sciences, University of Messina, Messina, Italy

Vera Osipova and her co-workers, on behalf of the Russian Headache Research Society, took one of our recent articles [1] as a starting point to express their opinion on the issue of chronic migraine (CM)/transformed migraine (TM) [2].

We are very grateful to them, because we think that this complex topic is of fundamental importance not only in terms of nosography and classification but even more so for its significant repercussions on research and clinical practice. We do believe that only through a wide-open and robust debate can we find a convincing explanation and achieve a shared consensus on the issue.

However, after a careful reading of our Russian colleagues’ opinions, we are left with the impression that there has been some misunderstanding, not only lexical but also conceptual. To sort out the question, let us try to proceed by steps starting from a strictly clinical consideration which we think all of us agree on.

The primary headache patients we are dealing with here, who have not yet found an adequate place in the existing classification systems, are those patients who have suffered for years from migraine without aura (MO) with a frequency of attacks varying usually between once a month and once a week. Then, this frequency progressively increases until there are no more free intervals between one attack and the next. In most such cases, there are also variations in the headache’s clinical features, such as reduction of the accompanying symptoms and changes in pain site. A considerable number of these patients eventually make excessive use of symptomatic medication.

Also from the clinical perspective, we believe that by now we should all agree on the two following considerations:

1. In these patients, there are two subsequent levels of severity that should be kept entirely separate: the first level (L1) is represented by MO with a very high frequency of attacks, but with clinical features that still fully match the diagnostic criteria of the ICHD-II [3] for MO; the second level (L2) is represented by a type of headache that has become chronic daily or almost daily and can be considered a true complication of MO.
2. L1 patients and especially L2 patients often make excessive use of symptomatic drugs, but most of the times the role (cause or effect) that these drugs play in the unfavourable evolution of headache is not clear.

Difficulties, and differences of opinion, arise when attempts are made at providing a terminological and descriptive systematization of such type(s) of patients.

The IHS classification of 1988 [4] included MO, but avoided dealing with both L1 and L2 patients. The ICHD-II [3] included CM among complications of migraine, but for this migraine subtype it provided diagnostic criteria that have not much correspondence with clinical practice, as they merely represent a fraction of L1 patients.

The ICHD-IIR [5] revised the diagnostic criteria of CM. The reworded criteria may now represent a large part of L2 patients, but certainly not L1 patients.

Our proposal [1] was aimed at systematizing both L1 and L2 patients by providing the respective diagnostic criteria for their headache. L1 patients were placed among MO subtypes, and L2 patients were placed among complications of migraine.

If we accept the clinical considerations expounded above, then we would be left with just one more problem to solve: what name should be given to L1 and L2 patients, respectively. In our opinion, this is a question that concerns the formal aspects of language and lexicon and therefore should not stand in the way of systematization efforts for headache subtypes that by now all clinicians basically agree on. Yet, today it is just the unsolved terminological question that still creates confusion and misunderstanding.

As L1 patients are migraineurs with a high frequency of attacks but do not have yet complicated migraine, in previous articles [6, 7] we had proposed for them the term “very frequent MO”, which we still find is the most appropriate and least misleading. In our article [1] commented on by Osipova et al. [2], we replaced “very frequent MO” with CM, following the suggestions by two referees who thought it was not advisable to abandon a name, like CM, that has already entered in common usage.

In our opinion, L2 patients are genuine complicated migraine sufferers and for them the name TM seems more appropriate.

TM is clearly more severe than “very frequent MO” or CM and this is reflected in the diagnostic criteria that we provided for the two migraine forms [1].

Therefore, we totally agree with Bigal et al. [8], who consider CM an early stage of TM. On the other hand, we find it surprising that in their comment, Osipova et al. [2] cited precisely Bigal et al. [8] to state that “chronification is a terminal stage of transformation”.

Such misunderstandings are certainly based on subtle differences in language, but the term CM is ambiguous, as Seshia et al. [9] and Olesen [10] himself recognized, and surely does not help in overcoming them. While “very frequent MO” seems to us a more appropriate term to describe L1 patients, the term CM could nonetheless be maintained out of convention, provided we clearly specify that, when used for migraine, the adjective “chronic” indicates a type of migraine with a high frequency of attacks.
